# MRI-assessed structural damage progression in sacroiliac joints of European routine care patients with axial spondyloarthritis receiving biological or conventional therapy

**DOI:** 10.1016/j.ero.2026.100269

**Published:** 2026-11-03

**Authors:** Annik Steimer, Jonas Brändli, Raphael Micheroli, Anna E.F. Hadsbjerg, Simon Krabbe, Nora Vladimirova, Iris Eshed, Torsten Diekhoff, Robert G.W. Lambert, Susanne Juhl Pedersen, Manouk de Hooge, Kristýna Bubová, Monika Gregová, Michael J. Nissen, Burkhard Möller, Claudia Lourenço Rodrigues, Jakub Závada, Ziga Snoj, Karlo Pintaric, Bjorn Gudbjornsson, Ziga Rotar, Iwona Sudol-Szopinska, Kasper Gosvig, Jakob M. Møller, Marie Wetterslev, Stephanie Wichuk, Merete Lund Hetland, Lykke M. Ørnbjerg, Mikkel Østergaard, Adrian Ciurea

**Affiliations:** 1 Department of Rheumatology, University Hospital of Zurich, University of Zurich, Zurich, Switzerland; 2 Swiss Clinical Quality Management in Rheumatic Diseases Foundation, Zurich, Switzerland; 3 Copenhagen Center for Arthritis Research, Center for Rheumatology and Spine Diseases, Centre for Head and Orthopaedics, Rigshospitalet, Glostrup, Denmark; 4 Department of Internal Medicine, Faculty of Health and Medical Sciences, University of Copenhagen, Copenhagen, Denmark; 5 Department of Diagnostic Imaging, Sheba Medical Center, Tel Hashomer affiliated with the Gray School of Medicine, Tel Aviv University, Tel Aviv, Israel; 6 Department of Radiology, Immanuel Clinic Rüdersdorf, University Hospital of Brandenburg Medical School, Rüdersdorf bei Berlin, Brandenburg, Germany; 7 Department of Radiology and Diagnostic Imaging, University of Alberta, Edmonton, AB, Canada; 8 Department of Rheumatology, Ghent University, Ghent, Belgium; 9 Department of Rheumatology and Institute of Rheumatology, First Faculty of Medicine Charles University, Prague, Czech Republic; 10 Rheumatology Department, Geneva University Hospital, Geneva, Switzerland; 11 Department of Rheumatology and Immunology, Inselspital, University Hospital Bern, Bern, Switzerland; 12 Institute of Radiology, UMC Ljubljana, Ljubljana, Slovenia; 13 Centre for Rheumatology Research, Landspitali University Hospital, Reykjavik, Iceland; 14 Faculty of Medicine, University of Iceland, Reykjavik, Iceland; 15 Department of Rheumatology, University Medical Centre Ljubljana, Ljubljana, Slovenia; 16 Faculty of Medicine, University of Ljubljana, Ljubljana, Slovenia; 17 Department of Radiology, National Institute of Geriatrics, Rheumatology and Rehabilitation, Warsaw, Poland; 18 Department of Radiology, Copenhagen University Hospital Herlev-Gentofte, Herlev, Denmark; 19 Division of Rheumatology, University of Alberta, Edmonton, AB, Canada

## Abstract

**Objectives:**

To describe progression of magnetic resonance imaging (MRI)–detected sacroiliac joint (SIJ) structural lesions in patients with axial spondyloarthritis (axSpA) in routine care.

**Methods:**

Bionaïve patients with axSpA from 5 European countries were included if a follow-up SIJ MRI was available 1 to 4 years after baseline. MRIs were centrally assessed using the Spondyloarthritis Research Consortium of Canada SIJ scoring systems by readers blinded to chronology. Patients were stratified by initiation of biological therapy during follow-up. Structural progression was described and compared.

**Results:**

Of 179 patients, 122 had a first MRI indicative of SpA. In these patients, baseline scores for bone marrow oedema (BME), fat lesions, and erosions were significantly higher in those initiating biologic disease-modifying antirheumatic drugs (bDMARDs) (n = 53) than in conventionally treated patients (n = 59). The MRI interval was 1.8 years in bDMARD-treated patients and 2.3 years in conventionally treated patients. In bDMARD-treated patients, mean Spondyloarthritis Research Consortium of Canada changes during MRI follow-up were BME −14.7, fat lesions +1.2, erosions −2.4, backfill +1.6, and ankylosis +0.2. Corresponding changes in conventionally treated patients were −3.0, +0.03, −0.04, +0.9, and +0.7. Ankylosis progression occurred in 15% of conventionally treated patients and 9% of those receiving bDMARDs. Backfill and ankylosis at baseline correlated with ankylosis progression in conventionally treated patients (Spearman’s ρ = 0.38 and 0.42) but not in bDMARD-treated patients (ρ = −0.17 and 0.09).

**Conclusions:**

Erosions decreased only with bDMARD therapy. Baseline backfill was associated with ankylosis progression in conventional but not bDMARD-treated patients, suggesting a potential dissociation between backfill and ankylosis under biologics. Future studies should assess causality.

**WHAT IS ALREADY KNOWN ON THIS TOPIC**

• Sacroiliac joint (SIJ) erosions detected by magnetic resonance imaging (MRI) following inflammation in axial spondyloarthritis may either resolve or be refilled with a tissue that is bright on T1-weighted MRI sequences (‘backfill’), which is considered an intermediate stage in the pathway to ankylosis.
• Post hoc analyses of randomised controlled trials (RCTs) of biologic or targeted synthetic disease-modifying antirheumatic drugs (b/tsDMARDs) suggest that these therapies may accelerate the sequence of structural SIJ lesion evolution.

**WHAT THIS STUDY ADDS**

• The pattern of structural SIJ changes observed in RCTs of b/tsDMARDs can be reproduced in a real-world treatment setting.
• The previously reported association between backfill and subsequent progression to ankylosis was not observed in patients receiving bDMARD therapy.

**HOW THIS STUDY MIGHT AFFECT RESEARCH, PRACTICE OR POLICY**

• Increases in backfill associated with bDMARD treatment may not necessarily lead to increased ankylosis.
• A more refined characterisation of imaging features reflecting different stages of bone damage and repair is needed to better understand the relationships between backfill and ankylosis.

Alt-text: Unlabelled box dummy alt text

## INTRODUCTION

The issue of disease modification in axial spondyloarthritis (axSpA), particularly the possibility of inhibiting—or at least decelerating—new bone formation at the level of the sacroiliac joints (SIJs) and the spine, remains a matter of ongoing debate [[1], [2], [3], [4]]. The placebo-controlled phases of randomised controlled trials (RCTs) of biologic or targeted synthetic disease-modifying antirheumatic drugs (b/tsDMARDs) are generally too short to detect effects on spinal bone formation. In contrast, several observational studies have shown, despite important methodological challenges, which bDMARD therapy may inhibit spinal radiographic progression [[5], [6], [7], [8], [9]]. Other cohort analyses have further indicated that tumour necrosis factor inhibitors (TNFi) may also decelerate radiographic progression at the level of the SIJs [10,11]. Recent magnetic resonance imaging (MRI) follow-up assessments from RCTs of b/tsDMARDs in axSpA have demonstrated rapid remodelling of SIJ tissues after treatment, characterised by reductions in erosions and increases in fat lesions and backfill [[12], [13], [14], [15], [16]]. As fat lesions and backfill are considered intermediate stages in the pathway towards ankylosis [17,18], it remains unclear whether their rapid increase following initiation of b/tsDMARD therapy could ultimately promote ankylosis. To date, only minimal increases in SIJ ankylosis have been observed over the relatively short follow-up periods (up to 52 weeks) of published RCTs [15,16]. Long-term follow-up data from observational cohort studies, which might help resolve this conundrum, remain sparse [[18], [19], [20]].

The aim of this study was to characterise the progression of MRI-detected SIJ lesions during routine clinical care in European patients with a clinical diagnosis of axSpA who either initiated bDMARD treatment or continued conventional therapy.

## METHODS

### Patients

The study is embedded within the framework of an imaging project conducted by the large European Spondyloarthritis Research Collaboration Network (EuroSpA) [21]. This initiative comprises imaging data from patients in 5 nationwide rheumatology registries: DANBIO (Denmark), SCQM (Switzerland), ATTRA (Czech Republic), biorx.si (Slovenia), and ICEBIO (Iceland). The overall project aims to analyse disease patterns in axSpA and axial psoriatic arthritis and to evaluate the value of imaging for outcome prediction and monitoring in routine clinical care, using radiographs and MRIs of the SIJs and the spine collected in a multinational patient cohort.

For this specific study, patients clinically diagnosed with axSpA by board-certified rheumatologists were included if they had at least 2 SIJ MRIs, with a follow-up MRI within 1 to 4 years of the first MRI, regardless of whether they were receiving bDMARDs or conventional treatment. MRIs should include both a semicoronal short tau inversion recovery or T2-weighted fat-saturated sequence and a semicoronal T1-weighted sequence without fat saturation. To ensure greater homogeneity within the study population, patients who were not b/tsDMARD-naive at the time of the first MRI or who had a first MRI before 2005 were excluded. Among patients who initiated a b/tsDMARD after the first MRI, those with short treatment duration (<14 weeks) or late treatment initiation (>9 months after the baseline MRI) were also excluded. Patient and disease characteristics, including treatment data, were extracted from the registries. B/tsDMARD-naive patients who did not initiate b/tsDMARD therapy during the entire observation period were classified as receiving conventional therapy (ie, nonsteroidal anti-inflammatory drugs, physical therapy, and/or analgesics). Patients initiating b/tsDMARDs could receive concomitant conventional therapy.

### Central SIJ scoring methodology

Central MRI assessment was performed by 2 calibrated readers experienced in MRI assessment of spondyloarthritis (SpA). They were blinded to all imaging and clinical information except sex. First, readers provided global evaluations of the first MRI using a standardised questionnaire, including whether the SIJ MRI was overall indicative of SpA, whether it fulfilled the Assessment of SpondyloArthritis International Society (ASAS) definition of a positive MRI (ie, active sacroiliitis), and whether findings were suggestive of alternative conditions [22]. In cases of disagreement between the 2 primary readers regarding whether an MRI was indicative of SpA, whether they fulfilled the ASAS MRI criteria for a positive MRI, adjudication was performed by an expert radiologist who is a member of the ASAS MRI Working Group [22]. Adjudication was also performed when predefined differential diagnoses (fracture, neoplasia, or infection) were identified by at least 1 reader.

For the subsequent central assessment of longitudinal MRI scans, images were read pairwise but in an unknown chronological order. MRIs were scored using the Spondyloarthritis Research Consortium of Canada (SPARCC) SIJ scoring system for inflammatory lesions (bone marrow oedema [BME] and structural lesions (erosions, fat lesions, backfill, and ankylosis) on 5 or 6 standardised semicoronal slices through the cartilaginous portion of the SIJs [23,24]. For the assessment of inflammatory and structural lesions using the SPARCC scoring system, no adjudication was performed.

### Statistical analyses

Status scores of the SPARCC SIJ inflammation index and for each structural lesion type, as well as change in scores from baseline to follow-up, were calculated as the average of the 2 readers.

Inter-reader reliability for SPARCC inflammatory and structural lesion scores was assessed using the intraclass correlation coefficient (ICC) based on a 2-way random-effects model for absolute agreement: ICC (2, k) at baseline and follow-up MRI, as well as for the change in scores [25]. The smallest detectable change (SDC) was also calculated as an additional assessment of inter-reader agreement. Progression and regression in ankylosis were defined using a prespecified threshold of 2 points. The SDC was reported separately to assess whether the observed changes exceeded the expected maximal measurement error. Spearman’s rank correlation coefficients were used for correlation analyses with the following interpretation of associations: 0 to 0.19 very weak, 0.2 to 0.39 weak, 0.4 to 0.59 moderate, 0.6 to 0.79 strong, and 0.8 to 1 very strong to perfect [26].

Baseline characteristics between patient groups were compared using the Kruskal-Wallis rank-sum test for continuous variables and Fisher’s exact test for categorical variables. Differences in SPARCC lesion scores between baseline and follow-up MRI scans were assessed using the Wilcoxon signed-rank test.

Direct statistical comparisons of the progression of different structural lesions between patients treated with bDMARDs and those treated conventionally were not performed, as causality between treatment and effects on progression cannot be established in the current investigational setting. Furthermore, a multivariable regression analysis was not feasible, because data on baseline disease activity (Ankylosing Spondylitis Disease Activity Score [ASDAS]), an important potential confounder, were unavailable in approximately half of the patients with an MRI indicative of SpA.

### Ethics

The study was conducted in accordance with the Helsinki Declaration. All participating registries had approval from the respective local ethics board and/or data protection agency for data collection, as required by national or local regulations prior to data transfer. In Denmark, the imaging project was approved by the Danish Patient Safety Authority, and ethics approval was waived. In Iceland, the National Bioethics Committee approved the project (18-009-V17). In Switzerland, the project and current study were approved by the Kantonale Ethikkommission, Kanton Zürich, BASEC 2022-00272. The National Medical Ethics Committee of the Republic of Slovenia approved the project (0120-97/2022). In the Czech Republic, ethical approval for ATTRA was granted by the Czech Multicentre Research Ethics Committee (No. 201611 S300). No additional ethical approval was required for the current analysis.

## RESULTS

### Patients characteristics

The disposition of patients included in the study is shown in Figure 1. Of the 309 patients with available MRI follow-up, 275 were bionaive at the time of the first scan. Patients were excluded when information regarding whether the first MRI was suggestive of SpA was unavailable. Among those who initiated bDMARD therapy, additional exclusions were applied to patients with short treatment exposure or late initiation of bDMARDs. Ultimately, 179 patients were retained for analysis. The mean (SD) interval between the 2 MRI scans was 2.1 (0.8) years. Interobserver reliability for detection of individual SPARCC inflammatory and structural lesions at baseline and follow-up MRI scans, as well as for detecting change in these domains, was poor to excellent (ICC, 0.31-0.96). Supplementary Table S1 shows ICCs and SDCs for the individual lesions.

**Figure 1:**
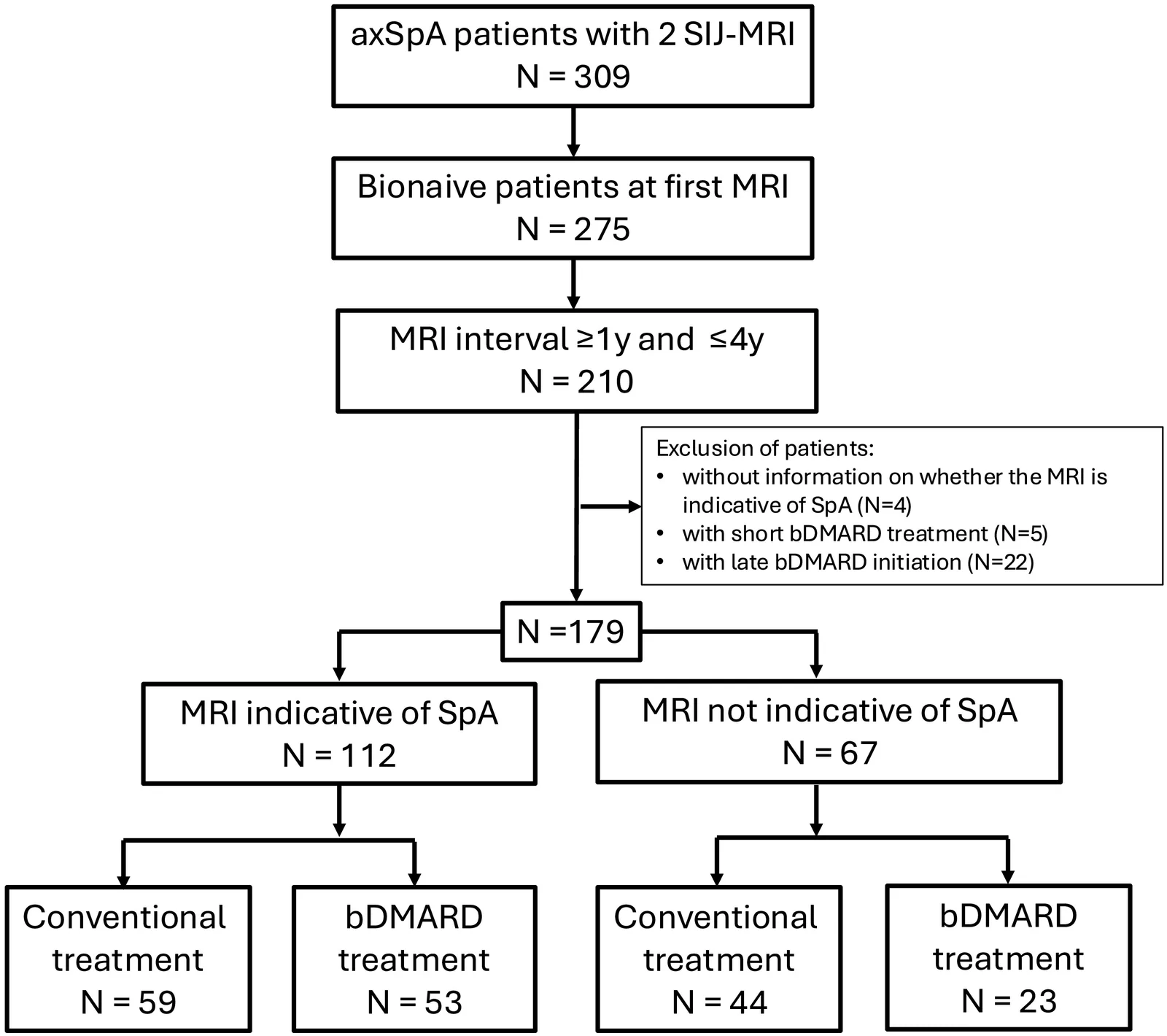
Disposition of patients with axial spondyloarthritis (axSpA) and available MRI follow-up within the European Spondyloarthritis Research Collaboration Network (EuroSpA). axSpA, axial spondyloarthritis; bDMARD, biologic disease-modifying antirheumatic drug; MRI, magnetic resonance imaging; N, number of patients; SIJ, sacroiliac joint; SpA, spondyloarthritis; y, year. Conventional therapy refers to treatment with nonsteroidal anti-inflammatory drugs, analgetics, and/or physical therapy.

Based on the overall judgement from central readings, 112 patients had a baseline MRI indicative of SpA, whereas 67 patients had an MRI not indicative of SpA. Baseline characteristics of these 2 groups are presented in Supplementary Table S2. Patients with a baseline MRI indicative of SpA were more frequently male (71% vs 28%) and human leukocyte antigen-B27 positive (79% vs 43%). A first bDMARD was initiated in 53 patients (47%) in the ‘MRI indicative of SpA’ group and in 23 patients (34%) in the ‘MRI not indicative of SpA’ group.

Baseline characteristics of patients who continued conventional therapy compared with those who received bDMARDs during the MRI interval are presented in Table 1, stratified by whether the baseline MRI was indicative of SpA. Among patients with an MRI indicative of SpA, those who initiated bDMARD treatment exhibited significantly higher levels of both subjective and objective markers of inflammation, including Bath Ankylosing Spondylitis Disease Activity Index, ASDAS, SPARCC SIJ BME scores, and C-reactive protein (Table 1). They also had greater SIJ structural damage, reflected by higher SPARCC erosion and fat lesion scores, whereas backfill and ankylosis scores were comparable between groups. The mean (SD) interval between MRIs was slightly longer in patients who continued conventional treatment than in those who initiated bDMARDs (2.3 [0.8] vs 1.8 [0.8] years, respectively). The median time from the first MRI to initiation of bDMARD therapy was 2 months, and the median duration of treatment (TNF or interleukin-17 inhibition) was 1.2 years, representing ∼80% of the interval between MRI scans. During follow-up, 40% of patients underwent a change in bDMARD therapy (Table 1). Baseline differences between conventional and bDMARD-treated patients were less pronounced among individuals with an MRI not indicative of SpA (Table 1).

**Table 1:** Baseline characteristics at first MRI of the sacroiliac joints in bDMARD-naïve patients, stratified by type of treatment during follow-up

	All patients N = 179		Baseline MRI indicative of SpA N = 112					Baseline MRI not indicative of SpA N = 67				
			Conventional treatment N = 59		bDMARD treatment N = 53			Conventional treatment N = 44		bDMARD treatment N = 23		
Country a	179		59		53		0.06	44		23		<0.001
• Denmark, N (%)		110 (61.5)		39 (66.1)		36 (67.9)			18 (40.9)		17 (73.9)	
• Czech Republic, N (%)		43 (24.0)		16 (27.1)		7 (13.2)			20 (45.5)		0 (0.0)	
• Switzerland, N (%)		25 (14.0)		4 (6.8)		10 (18.9)			5 (11.4)		6 (26.1)	
• Slovenia, N (%)		1 (0.6)		0 (0.0)		0 (0.0)			1 (2.3)		0 (0.0)	
Calendar period of first MRI	179		59		53		0.08	44		23		0.06
• 2005-2009		12 (6.7)		3 (5.1)		6 (11.3)			1 (2.3)		2 (8.7)	
• 2010-2014		46 (25.7)		17 (28.8)		6 (11.3)			18 (40.9)		5 (21.7)	
• 2015-2019		108 (60.3)		35 (59.3)		34 (64.2)			25 (56.8)		14 (60.9)	
• 2020-2024		13 (7.3)		4 (6.8)		7 (13.2)			0 (0.0)		2 (8.7)	
Time interval between MRIs, y	179	2.1 (0.8)	59	2.3 (0.8)	53	1.8 (0.8)	<0.001	44	2.2 (0.6)	23	1.8 (0.7)	0.051
Male sex, N (%)	179	99 (55.3)	59	43 (72.9)	53	37 (69.8)	0.83	44	14 (31.8)	23	5 (21.7)	0.57
Age, y	179	38.2 (11.0)	59	34.9 (9.0)	53	38.1 (11.1)	0.13	44	41.2 (12.1)	23	41.5 (10.8)	0.69
Symptom duration, y	137	8.2 (9.2)	46	7.5 (8.4)	41	9.1 (8.3)	0.18	35	8.7 (10.9)	15	6.9 (10.3)	0.16
HLA-B27 positive, N (%)	173	114 (65.9)	58	46 (79.3)	52	41 (78.8)	1.00	42	24 (57.1)	21	12 (57.1)	1.0
BASDAI	86	4.9 (2.4)	27	3.2 (2.1)	34	6.2 (1.9)	<0.001	14	4.5 (2.5)	11	5.7 (1.6)	0.24
ASDAS	82	3.1 (1.1)	25	2.5 (1.0)	33	3.8 (0.8)	<0.001	13	2.8 (1.1)	11	3.0 (0.7)	0.34
C-reactive protein, mg/L	91	11.4 (15.8)	30	8.0 (6.6)	35	17.6 (21.9)	0.06	14	9.4 (10.1)	12	4.6 (10.0)	0.12
EMM ever, N (%)	171	79 (46.2)	57	27 (47.4)	52	24 (46.2)	1.00	40	18 (45.0)	22	10 (45.5)	1.00
Current smoking, N (%)	177	58 (23.8)	58	17 (29.3)	53	19 (35.8)	0.77	44	13 (29.5)	22	9 (40.9)	0.22
Body mass index	141	25.4 (4.5)	36	24.1 (4.0)	51	25.5 (4.3)	0.13	34	25.8 (4.9)	21	26.5 (4.6)	0.54
ASAS-positive MRI (active sacroiliitis), N (%)	179	87 (48.6)	59	46 (78.0)	53	41 (77.4)	1.00	44	0 (0.0)	23	0 (0.0)	NA
Radiographic axSpA, N (%)	76	21 (27.6)	27	8 (29.6)	22	9 (40.9)	0.75	24	3 (12.5)	3	1 (33.3)	0.39
SPARCC BME score (range 0-72) median (IQR)	179	12.5 (13.6) 7.5 (1.5; 19.0)	59	14.8 (13.1) 12.5 (4.3; 24)	53	20.7 (15.5) 17.0 (9.0; 34.5)	0.047	44	3.5 (5.0) 1.5 (0.0; 5.1)	23	5.2 (5.2) 4.0 (1.3; 7.3)	0.11
SPARCC sacroiliac joints structural score												
• Erosion (range 0-40) median (IQR)	179	4.6 (5.1) 3.0 (0.5; 7.3)	59	5.5 (5.0) 4.5 (1.5; 8.3)	53	7.7 (5.7) 6.5 (3.5; 12.5)	0.04	44	1.1 (1.4) 1.0 (0.0; 1.0)	23	1.7 (2.5) 1.0 (0.0; 1.8)	0.50
• Fat lesion (range 0-40) median (IQR)	179	4.0 (6.0) 1.5 (0.0; 5.5)	59	4.0 (5.3) 2.0 (0.0; 6.5)	53	6.9 (8.1) 4.0 (0.5; 10.0)	0.048	44	0.8 (1.4) 0.0 (0.0; 1.0)	23	3.5 (3.9) 3.0 (0.3; 4.5)	<0.001
• Backfill (range 0-20) median (IQR)	179	1.1 (2.6) 0.0 (0.0; 0.8)	59	1.2 (1.9) 0.0 (0.0; 1.3)	53	2.2 (4.0) 0.0 (0.0; 2.5)	0.73	44	0.2 (0.9) 0.0 (0.0; 0.0)	23	0.1 (0.3) 0.0 (0.0; 0.0)	0.38
• Ankylosis (range 0-20) median (IQR)	179	1.1 (3.5) 0.0 (0.0; 0.0)	59	1.9 (4.7) 0.0 (0.0; 0.3)	53	1.4 (3.9) 0.0 (0.0; 0.0)	0.33	44	0.1 (0.6) 0.0 (0.0; 0.0)	23	0.0 (0.0) 0.0 (0.0; 0.0)	0.20
Number of different b/tsDMARDs during FU	179		59		53		NA	44		23		NA
• 1		43 (56.6)		0 (0)		32 (60.4)			0 (0)		11 (47.8)	
• 2		24 (31.6)		0 (0)		17 (32.1)			0 (0)		7 (30.4)	
• 3		9 (11.8)		0 (0)		4 (7.5)			0 (0)		5 (21.7)	
Modes of action of b/tsDMARD during FU	179		59		53		NA	44		23		NA
• Only TNF inhibition		52 (68.4)		0 (0)		36 (67.9)			0 (0)		16 (69.6)	
• Only IL-17 inhibition		10 (13.2)		0 (0)		9 (17.0)			0 (0)		1 (4.3)	
• Both TNF and IL-17 inhibition		14 (18.4)		0 (0)		8 (15.1)			0 (0)		6 (26.1)	
• Janus kinase inhibition		0 (0)		0 (0)		0 (0)			0 (0)		0 (0)	
Time on bDMARD during FU, years, median (IQR)	179	0.0 (0.0; 1.0)	59	0.0 (0.0; 0.0)	53	1.2 (1.0; 1.8)	NA	44	0.0 (0.0; 0.0)	23	1.0 (0.7; 1.5)	NA
Time to first bDMARD, years, median (IQR)	179	0.2 (0.0; 0.4)	59	0.0 (0.0; 0.0)	53	0.2 (0.0; 0.3)	NA	44	0.0 (0.0; 0.0)	23	0.2 (0.0; 0.4)	NA
Proportion of interval on bDMARD, median (IQR)	179	0.0 (0.0; 0.7)	59	0.0 (0.0; 0.0)	53	0.8 (0.7; 0.9)	NA	44	0.0 (0.0; 0.0)	23	0.6 (0.5; 0.8)	NA

ASAS, Assessment of SpondyloArthritis International Society; ASDAS, Ankylosing Spondylitis Disease Activity Score; axSpA, axial spondyloarthritis; BASDAI, Bath Ankylosing Spondylitis Disease Activity Index; b/tsDMARD, biologic or targeted synthetic disease-modifying antirheumatic drug; BME, bone marrow oedema; EMM, extra-musculoskeletal manifestations; FU, follow-up (MRI interval); HLA-B27, human leucocyte antigen B27; IL-17, interleukin-17; MRI, magnetic resonance imaging; SpA, spondyloarthritis; SPARCC, Spondyloarthritis Research Consortium of Canada; TNF, tumour necrosis factor.

Values are the mean (SD), except where indicated otherwise. Conventional therapy refers to treatment with nonsteroidal anti-inflammatory drugs, analgetics and/or physical therapy.

a No patients from Iceland fulfilled the inclusion criteria of this study.

### Changes in SPARCC MRI structural damage scores over 2 years

Table 2 presents the mean (SD) sum scores for individual SPARCC domains at baseline and follow-up MRI scans, as well as the mean (SD) and median (IQR) changes in these scores. Results are shown for patients with MRI findings indicative and not indicative of SpA, and stratified by treatment during the MRI interval (conventional therapy vs bDMARDs). As no relevant changes in SPARCC SIJ structural damage scores were observed in patients with a baseline MRI not indicative of SpA, subsequent analyses focus on the group with MRI findings indicative of SpA (Table 2). In patients treated with bDMARDs, a mean reduction of 14.7 units in the BME score was accompanied by a mean decrease of 2.4 units in the erosion score, along with increases of mean 1.2 units in the fat lesion score, mean 1.6 units in the backfill score, and mean 0.2 units in the ankylosis score. The corresponding changes in SPARCC scores among patients receiving conventional therapy were a decrease of 3.0 units for BME, 0.04 units for erosion, an increase of 0.9 units for backfill, and of 0.7 units for ankylosis (Table 2). The number of patients demonstrating progression or regression of ankylosis—defined as a change of at least 2 units in the SPARCC ankylosis domain, exceeding the SDC of 1.4—is presented in Table 3. Progression of ankylosis was observed in 9 patients (15%) receiving conventional therapy and in 5 patients (9%) treated with bDMARDs. Changes in SPARCC ankylosis sum scores are plotted against the interval between MRI scans in the Supplementary Figure S1. Progression, as defined above, was observed across all MRI follow-up intervals, ranging from 1 to 4 years in both conventionally treated patients and those receiving bDMARDs. Thus, although patients with ankylosis progression had a longer median interval between MRI examinations (2 vs 2.8 years), progression was observed across both shorter and longer follow-up periods, ie, the observed progression was not confined to patients with longer MRI intervals.

**Table 2:** Baseline and change in SPARCC sum scores for the indicated domains on SIJ MRI in patients treated either conventionally or with bDMARDs

		Baseline MRI indicative of SpA					Baseline MRI not indicative of SpA				
Treatment	SPARCC domain	Sum score at BL mean (SD)	Sum score at FU mean (SD)	SPARCC change mean (SD)	SPARCC change median (IQR)	*P* value	Sum score at BL mean (SD)	Sum score at FU mean (SD)	SPARCC change mean (SD)	SPARCC change median (IQR)	*P* value
Conventional	BME (range 0-72)	14.8 (13.1)	11.9 (13.4)	–3.0 (14.6)	–2.0 (–8.0; 1.8)	.07	3.5 (5.0)	3.3 (4.7)	–0.1 (4.8)	0.0 (–0.6; 1.0)	.89
	Erosion (range 0-40)	5.5 (5.0)	5.5 (5.5)	–0.04 (3.5)	0.0 (–2.5; 1.75)	.75	1.1 (1.4)	0.9 (1.3)	–0.2 (0.9)	0.0 (–0.5; 0.6)	.26
	Fat lesion (range 0-40)	4.0 (5.3)	4.1 (5.6)	0.03 (3.1)	0.0 (–1.0; 0.3)	.60	0.8 (1.4)	1.1 (2.1)	0.4 (1.8)	0.0 (0.0; 0.0)	.46
	Backfill (range 0-20)	1.2 (1.9)	2.0 (2.9)	0.9 (2.6)	0.0 (0.0; 1.0)	.01	0.2 (0.9)	0.5 (1.5)	0.2 (0.7)	0.0 (0.0; 0.0)	.01
	Ankylosis (range 0-20)	1.9 (4.7)	2.6 (5.5)	0.7 (2.1)	0.0 (0.0; 0.0)	.01	0.1 (0.6)	0.1 (0.6)	0.0 (0.4)	0.0 (0.0; 0.0)	1.0
bDMARDs	BME (range 0-72)	20.7 (15.5)	6.0 (10.4)	–14.7 (17.4)	–11.5 (–27.5; –1.0)	<.001	5.2 (5.2)	3.2 (3.9)	–2.0 (4.9)	0.0 (–3.0; 0.0)	.09
	Erosion (range 0-40)	7.7 (5.7)	5.2 (3.8)	–2.4 (5.1)	–2.0 (–5.0; 0.0)	<.001	1.7 (2.5)	1.2 (1.5)	–0.5 (1.8)	0.0 (–0.5; 0.0)	.36
	Fat lesion (range 0-40)	6.9 (8.1)	8.1 (7.3)	1.2 (3.6)	1.0 (–0.5; 3.5)	.01	3.5 (3.9)	3.5 (4.2)	0.1 (1.9)	0.0 (–0.5; 0.8)	.89
	Backfill (range 0-20)	2.2 (4.0)	3.7 (4.7)	1.6 (3.4)	0.5 (0.0; 2.0)	<.001	0.1 (0.3)	0.4 (0.9)	0.3 (0.8)	0.0 (0.0; 0.0)	.20
	Ankylosis (range 0-20)	1.4 (3.9)	1.6 (4.1)	0.2 (1.2)	0.0 (0.0; 0.0)	.23	0.0 (0.0)	0.0 (0.0)	0.0 (0.0)	0.0 (0.0; 0.0)	NA

bDMARDs, biologic disease-modifying antirheumatic drugs; BL, baseline (first) MRI; BME, bone marrow oedema; FU, follow-up MRI; MRI, magnetic resonance imaging; SIJ, sacroiliac joint; SPARCC, Spondyloarthritis Research Consortium of Canada.

Conventional therapy refers to treatment with nonsteroidal anti-inflammatory drugs, analgetics and/or physical therapy.

**Table 3:** Categorised change in SPARCC ankylosis score as defined by a change above the smallest detectable change, corresponding to a change of at least 2 units^a^

Baseline MRI indicative of SpA	Treatment	N	Progression (≥2) N (%)	Change of –2 to 2 N (%)	Regression (≤–2) N (%)
yes	conventional	59	9 (15.3)	50 (84.7)	0 (0.0)
	bDMARDs	53	5 (9.4)	46 (86.8)	2 (3.8)
no	conventional	44	0 (0.0)	43 (97.7)	1 (2.3)
	bDMARDs	23	0 (0.0)	23 (100.0)	0 (0.0)

bDMARDs, biologic disease-modifying antirheumatic drugs; MRI, magnetic resonance imaging; SpA, spondyloarthritis; SPARCC, Spondyloarthritis Research Consortium of Canada.

a Conventional therapy refers to treatment with nonsteroidal anti-inflammatory drugs, analgetics, and/or physical therapy. The smallest detectable change for ankylosis in this study was 1.4.

### Correlations between baseline scores and subsequent changes in different SPARCC domains

Figure 2 presents correlation matrices illustrating the relationships between baseline SIJ lesions and subsequent changes in inflammatory and structural MRI lesions during follow-up, stratified by treatment (conventional therapy vs bDMARDs). Baseline BME was moderately associated with a reduction in BME and weakly associated with a progression of fat lesions and backfill during follow-up in patients treated conventionally. Among bDMARD-treated patients, baseline BME scores showed a very strong association with their subsequent reduction, as well as moderate and weak associations with progression of fat lesions and backfill, respectively. In the bDMARD treatment group, baseline erosions were strongly associated with their subsequent regression and were also moderately associated with progression of backfill. Baseline erosions were weakly associated with erosion regression at follow-up in patients receiving conventional treatment. Furthermore, baseline fat lesions, backfill, and ankylosis demonstrated weak to moderate associations with subsequent progression of ankylosis in conventionally treated patients. However, these associations with the development of ankylosis were not observed in patients treated with bDMARDs during the MRI follow-up period. Neither baseline bone marrow oedema nor erosions were associated with the development of ankylosis in conventionally or biologically treated patients (Fig 2).

**Figure 2:**
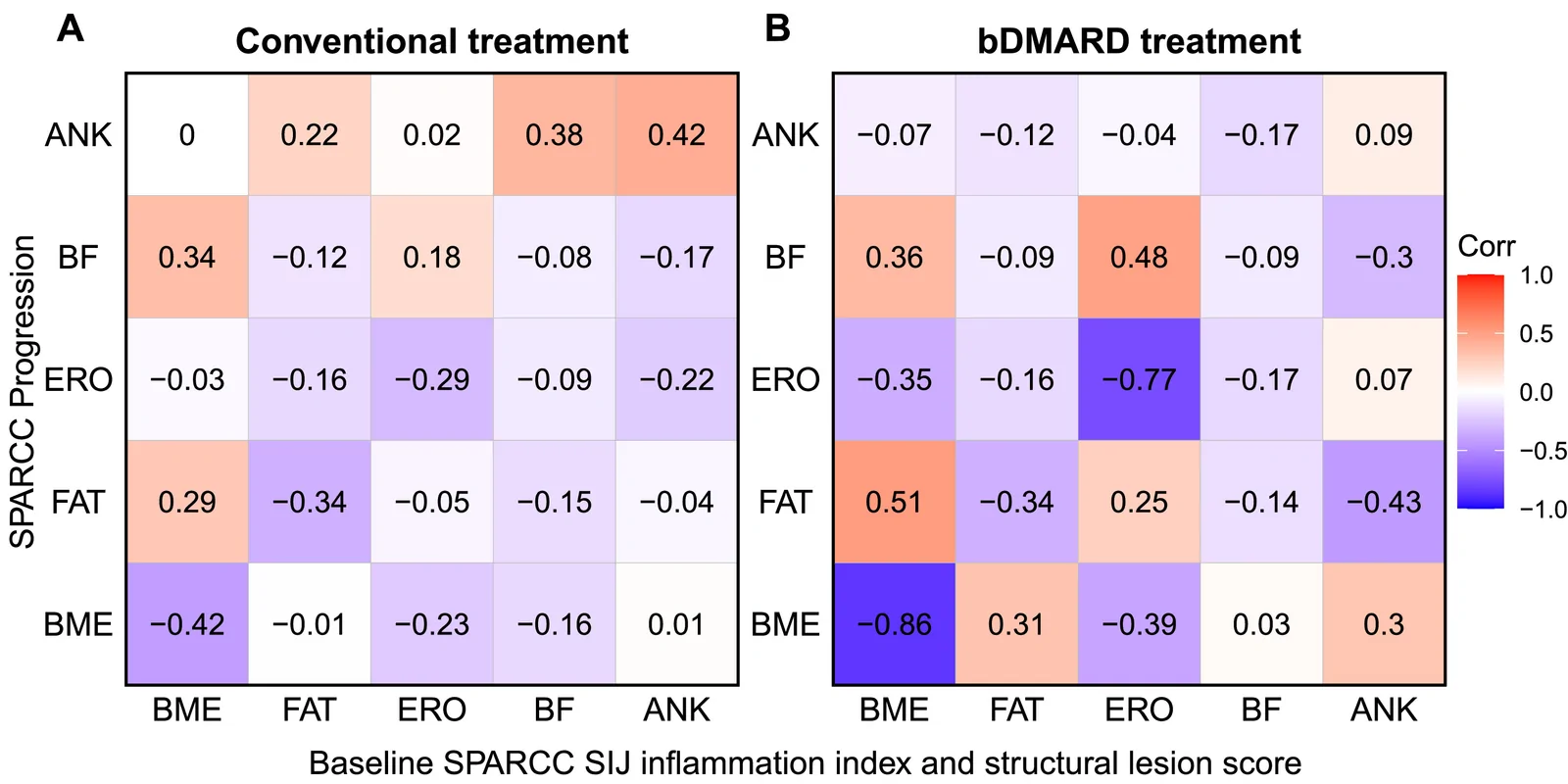
Correlation matrix demonstrating associations between SPARCC sacroiliac inflammation index (BME score) and structural lesion scores at baseline on the x-axis and the progression of individual sacroiliac joint lesions on the y-axis in patients with baseline MRI indicative of SpA. (A) Correlation in bionaive patients on conventional treatment during the MRI interval. (B) Correlations in patients treated with a first bDMARD during the MRI interval. Spearman’s rank correlation is used with the following interpretation of coefficients: 0 to 0.19 very weak association; 0.2 to 0.39 weak association; 0.4 to 0.59 moderate association; 0.6 to 0.79 strong association; and 0.8 to 1 very strong association. ANK, ankylosis; axSpA, axial spondyloarthritis; bDMARD, biologic disease-modifying antirheumatic drug; BF, backfill; BME, bone marrow oedema; ERO, erosions; FAT, fat lesions; MRI, magnetic resonance imaging; SIJ, sacroiliac joint; SpA, spondyloarthritis; SPARCC, Spondyloarthritis Research Consortium of Canada. Conventional therapy refers to treatment with nonsteroidal anti-inflammatory drugs, analgetics, and/or physical therapy.

Figure 3 presents scatter plots comparing baseline fat lesions or backfill with subsequent ankylosis progression during follow-up at the individual patient level, stratified by treatment group. Although patients receiving conventional therapy had lower baseline fat lesion and backfill scores, they exhibited greater increases in ankylosis scores compared with patients treated with bDMARDs.

**Figure 3:**
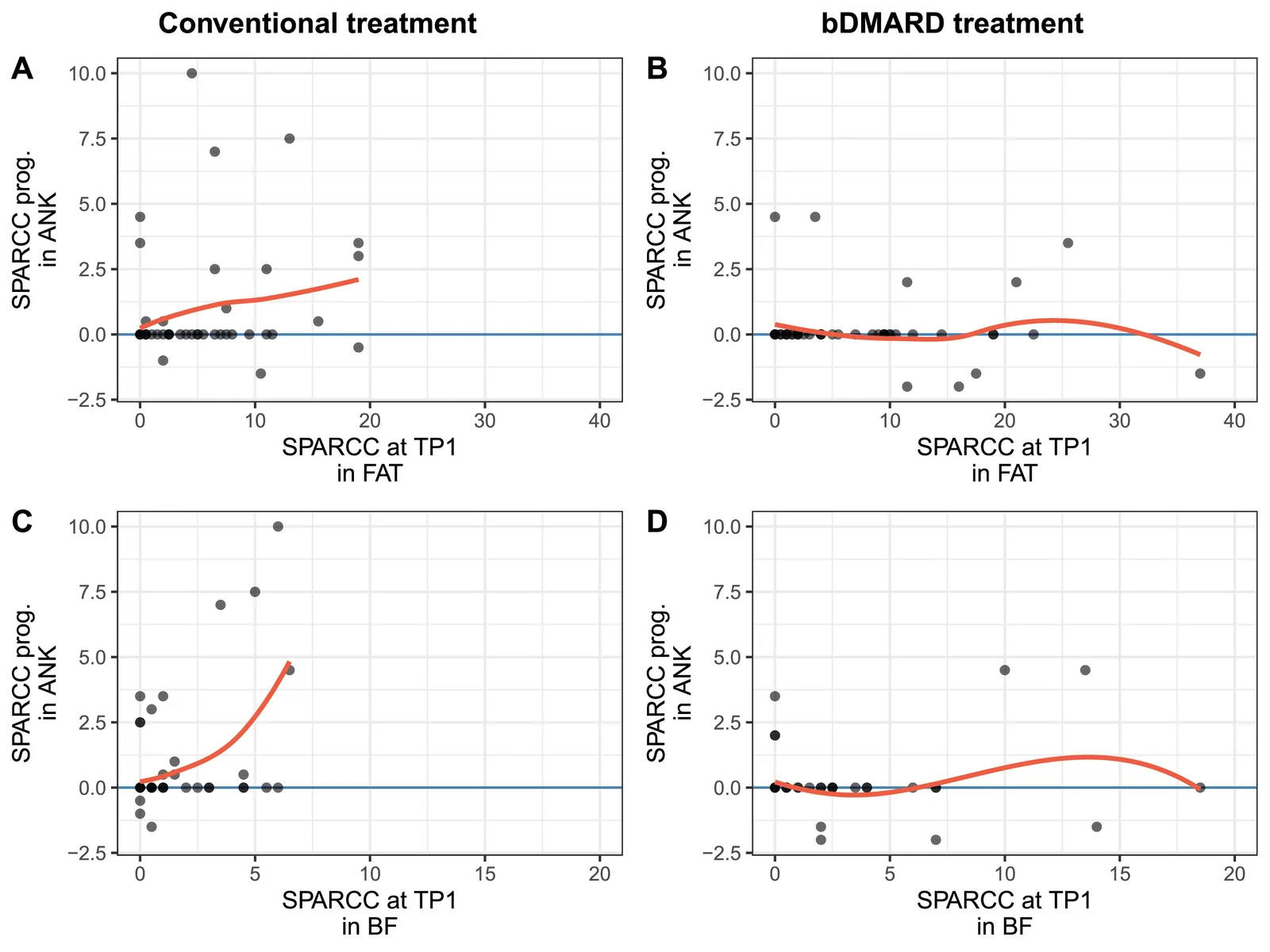
Scatter plots comparing baseline SPARCC sacroiliac joint structural lesion score for either fat lesions (A,B) or backfill (C,D) with progression of the SPARCC sacroiliac joint structural lesion score for ankylosis during the MRI interval in patients with a baseline MRI indicative of SpA. (A,C) Bionaive patients on conventional treatment during the MRI interval. (B,D) Patients on a first bDMARD during the MRI interval. A red smoothing line (local polynomial regression [LOESS] with parameter α = 1) is laid over the data. Conventional therapy refers to treatment with nonsteroidal anti-inflammatory drugs, analgetics, and/or physical therapy. ANK, ankylosis; bDMARD, biologic disease-modifying antirheumatic drug; BF, backfill; FAT, fat lesions; MRI, magnetic resonance imaging; SpA, spondyloarthritis; SPARCC, Spondyloarthritis Research Consortium of Canada; TP1, First timepoint.

### Correlations between changes in SPARCC MRI structural lesions

Two-year changes in BME showed weak to moderate correlations with changes in erosion and were negatively correlated with changes in fat lesions and backfill (Supplementary Fig S2). Progression in fat lesions correlated weakly to moderately with progression in backfill, whereas progression in erosions was negatively associated with progression in backfill.

## DISCUSSION

In this real-world European collaborative study of patients with axSpA undergoing longitudinal SIJ MRI assessment, treatment with bDMARDs was associated with marked reductions in inflammation and erosions, alongside increases in fat lesions and backfill, whereas progression to ankylosis remained limited. Importantly, the previously reported association between backfill and subsequent ankylosis progression was confirmed in conventionally treated patients but was not observed in those receiving bDMARD therapy. These findings suggest that treatment-related structural remodelling processes, including the development of fat lesions and backfill, do not necessarily translate into increased ankylosis in the context of biological therapy. Furthermore, the pattern of structural SIJ changes previously reported in RCTs of b/tsDMARDs could be replicated in routine clinical care over a longer MRI follow-up period.

Post-hoc analyses of several RCTs suggest that b/tsDMARD may accelerate the sequence of SIJ lesion development observed in axSpA, whereby BME may resolve, evolve into fat lesions, or progress to bone erosion, which can either resolve or subsequently develop into backfill, ie, become refilled with a tissue that is bright on T1-weighted MRI sequences [[12], [13], [14], [15], [16]]. In our real-world cohort of patients treated with bDMARDs, we observed a comparable pattern of tissue response, with positive correlations between the presence of BME or erosions at baseline and the subsequent development of fat lesions and backfill and an inverse correlation between changes in erosion scores and changes in backfill.

From a descriptive perspective, changes in structural lesions over a period of 2.3 years were overall more limited in patients receiving conventional therapy. In this group, correlations between baseline BME or erosions and subsequent progression of fat lesions and backfill were weaker. Consistent with the concept that fat lesions and backfill represent intermediate stages in the pathway towards ankylosis, moderate correlations were observed between baseline SPARCC scores for fat, backfill, and ankylosis and subsequent development of ankylosis in this group. In contrast, such associations could not be demonstrated in patients receiving bDMARD therapy. The observation period was slightly longer in conventionally treated patients (mean 2.3 vs 1.8 years), potentially allowing more time for ankylosis to develop. However, ankylosis progression occurred across time intervals ranging from 1 to 4 years in both treatment groups, suggesting that additional mechanisms may be involved. A potential dissociation between earlier structural lesions—such as fat lesions and backfill—and the final damage state, namely ankylosis, during bDMARD treatment may represent a plausible, nonmutually exclusive explanation.

The clinical implications of our study are multifaceted and may inform future research directions. The observed reductions in SIJ BME are consistent with the well-established ability of bDMARDs to effectively control inflammatory disease activity. Under bDMARD therapy, the presence of fat lesions and backfill was not associated with marked ankylosis progression during follow-up, alleviating concerns regarding potential accelerated osteoproliferative processes. Although this study does not permit causal inference, it supports the generation of hypotheses. The apparent disruption of the canonical sequence during bDMARD therapy may indicate a shift towards a reparative process, contrasting with the progressive chain of structural damage that culminates in ankylosis. Therefore, the suppression of new inflammatory lesions by bDMARD therapy may contribute to the lack of subsequent new bone formation, despite the development of additional fat lesions and backfill. Future investigations are needed to better elucidate the relationship between individual structural lesions during bDMARD therapy. It is conceivable that distinct stages of backfill exist, and that only specific stages—potentially defined by the extent or pattern of matrix calcification—are associated with subsequent ankylosis formation. A more refined characterisation of imaging features reflecting different stages of bone damage and repair may therefore be required to clarify the relationships between backfill and ankylosis [17]. Moreover, given the relatively short follow-up period, the absence of substantial ankylosis progression in the current study does not exclude a potential risk of ankylosis development over a longer period.

The choice of imaging modality for assessing progression of structural lesions at the level of the SIJs was largely dictated by the availability of MRI examinations obtained during routine clinical care across the participating European registries. MRI was also particularly relevant to the aims of the current study, as it permits simultaneous assessment of inflammatory and structural lesions and their temporal relationship. However, as MRI is less sensitive than computed tomography (CT) for the detection of ankylosis, the use of CT imaging may have yielded different results. Future studies incorporating CT-based assessment of the SIJs could further investigate the progression of ankylosis and its relationship with inflammatory and structural MRI lesions. The advent of MRI-based synthetic CT may be of particular interest in this regard.

We deliberately refrained from estimating differences in the progression of structural SIJ changes between patients treated with bDMARDs and those receiving conventional therapy for several important reasons. First, treatment allocation was strongly influenced by patient characteristics, as expected based on international recommendations for the management of axSpA [27]. Consequently, patients initiating bDMARD therapy had substantially higher disease activity and greater structural damage at baseline. Although these factors would be expected to lead to greater structural progression in the bDMARD group rather than the opposite, potential selection bias between treatment groups cannot be excluded. Second, the comparator group receiving conventional treatment was not well defined within this dataset, as detailed information on nonsteroidal anti-inflammatory drug use and engagement in physical therapy was not available. Third, in real-world clinical practice, the indications for obtaining follow-up MRI typically differ between patients treated with bDMARDs and those managed with conventional therapy. Finally, MRI assessments were not consistently synchronised with clinical visits, resulting in incomplete and likely nonrandomly missing data on disease activity, a key potential confounder.

Several additional limitations should be acknowledged. Patients with axSpA in real-world settings may represent a more heterogeneous population than those enrolled in clinical trials and may include individuals who have been misdiagnosed. In this context, current treatment guidelines recommend reconsidering the diagnosis in patients who do not respond adequately to bDMARD therapy [27]. To mitigate this limitation, we conducted the main analyses in a subgroup of patients with SIJ MRI findings indicative of SpA [22], as determined by central reading, thereby aligning our approach with the inclusion criteria typically applied in RCTs. A further limitation is that MRI data were not prospectively collected in all patients within the registries; instead, patients with available MRI scans were retrospectively identified at participating centres. Finally, information on physical activity, type of occupation, and childbirth—all factors that could affect MRI interpretation—was not available.

Strengths of our investigation include inclusion of patients from different European countries and the central reading of all MRIs by experienced and calibrated readers.

In conclusion, our investigation of patients with axSpA and a longer MRI follow-up period confirms that the SIJ structural changes observed in RCTs of b/tsDMARDs are also evident in real-world clinical practice and additionally suggests that treatment-associated increases in backfill may not necessarily translate into increased ankylosis.

## Competing interests

AEFH received grants from Novartis paid to the employer. AS received a grant from the MLR Foundation. BM received travel expenditures and honoraria for lectures or presentations from AbbVie, Janssen, Novartis and Pfizer. CLR received a grant from Novartis paid to the employer. IE received honoraria for lectures or presentations from Novartis. JB received a grant from Novartis paid to the employer. JZ received honoraria for lectures from AbbVie, Sobi, and UCB. KB received honoraria for lectures or presentations from Novartis, AbbVie, Eli Lilly, Pfizer, and Janssen. LMØ received grants from Novartis, UCB, and AbbVie paid to the employer. MdH received honoraria for presentations from UCB and Adium. MG received honoraria for lectures or presentations from Novartis, AbbVie, UCB, MSD, and Eli Lilly. MJN received honoraria for travel expenditures, lectures, or presentations from AbbVie, Eli Lilly, Janssen, Novartis, Pfizer, and UCB. MLH received consultancy fees from AbbVie (No personal income, paid to institution) and speaker fees from Pfizer, Medac, Sandoz (no personal income, institution) and from Novartis (personal income). Prev. chaired the steering committee of the Danish Rheumatology Quality Registry (DANBIO, DRQ), which receives public funding from the hospital owners and funding from pharmaceutical companies. Research grants (institution) from AbbVie, Biogen, BMS, Celltrion, Eli Lilly, Janssen Biologics B.V., Lundbeck Fonden, MSD, Medac, Pfizer, Roche, Samsung Biopies, Sandoz, Novartis, and Nordforsk. MØ received research grants from AbbVie, BMS, Merck, Novartis, and UCB and speaker and/or consultancy fees from AbbVie, BMS, Boehringer-Ingelheim, Celgene, Eli-Lilly, Galapagos, Gilead, Hospira, Janssen, MEDAC, Merck, Novartis, Novo, Orion, Pfizer, Regeneron, Roche, Sandoz, Sanofi, and UCB. NV received grants from Novartis paid to the employer. RGWL received consulting fees from AbbVie and CARE Arthritis Ltd. RM received honoraria for lectures or presentations from AbbVie, Janssen, and Eli Lilly. SJP received grants and contracts from Innovation Fund Denmark and Nordic Bioscience A/S and consulting fees, speaking fees, and/or research support from AbbVie, Novartis, MSD, Pfizer, and UCB. SK received grants from Novartis paid to the employer. TD received honoraria for lectures from Canon MS, Novartis, AbbVie, Bracco, and UCB and consulting fees from AbbVie and UCB. ZR received consulting fees from AbbVie, Novartis, Eli Lilly, Pfizer, Janssen, Sobi, Swixx BioPharma, and AstraZeneca and speakers’ fees from AbbVie, Amgen, Novartis, MSD, Medis, Biogen, Eli Lilly, Pfizer, Sanofi, Lek, and Janssen. ZS received honoraria for presentations from BioGen. All other authors declare they have no competing interests.

## Appendix Supplementary materials

Image, application 1

## Footnotes

Supplementary material associated with this article can be found in the online version at doi:10.1016/j.ero.2026.100269.
