# Supplementary material for: MRI-assessed structural damage progression in sacroiliac joints of European routine care patients with axial spondyloarthritis receiving biological or conventional therapy
Source: EULAR Rheumatol Open. 2026 Nov 3;2(4):100269. doi: 10.1016/j.ero.2026.100269 (PMC13756249; doi:10.1016/j.ero.2026.100269)
Supplement: Supplementary file 1 [file mmc1.pdf]

Supplementary Material

"Progression of MRI sacroiliac joint structural lesions over 2 years in axial spondyloarthritis: descriptive observational data from the EuroSpA Research Collaboration Network", by Steimer A. et al.

Supplementary Table S1. Interobserver reliability: Intraclass correlation coefficients and smallest detectable change for MRI scoring in the 179 included patients.

	Intraclass correlation coefficients ICC (2, k)			Smallest detectable change
SPARCC domain	Timepoint 1 (95% CI)	Timepoint 2 (95% CI)	Change from TP1 to TP2 (95% CI)	
Bone marrow oedema	0.91 (0.86; 0.94)	0.92 (0.89; 0.94)	0.94 (0.93; 0.95)	6.5
Structural lesions				
• Erosion	0.70 (0.61; 0.78)	0.58 (0.49; 0.65)	0.69 (0.55; 0.67)	4.5
• Fat lesion	0.55 (0.41; 0.66)	0.54 (0.39; 0.65)	0.31 (0.24; 0.38)	4.7
• Backfill	0.72 (0.66; 0.77)	0.66 (0.59; 0.72)	0.69 (0.64; 0.74)	2.6
• Ankylosis	0.94 (0.93; 0.96)	0.96 (0.95; 0.97)	0.72 (0.67; 0.77)	1.4

SPARCC = Spondyloarthritis Research Consortium of Canada.

Supplementary Table S2. Baseline characteristics at first sacroiliac joint MRI in bDMARD-naïve patients.

Parameter		All patients N = 179		Baseline MRI indicative of SpA N = 112		Baseline MRI not indicative of SpA N = 67	P
Country*	179		112		67		0.13
• Denmark, N (%)		110 (61.5)		75 (67.0)		35 (52.2)	
• Czech Republic, N (%)		43 (24.0)		23 (20.5)		20 (29.9)	
• Switzerland, N (%)		25 (14.0)		14 (12.5)		11 (16.4)	
• Slovenia, N (%)		1 (0.6)		0 (0.0)		1 (1.5)	
Calendar period of first MRI	179		112		67		0.09
• 2005-2009		12 (6.7)		9 (8.0)		3 (4.5)	
• 2010-2014		46 (25.7)		23 (20.5)		23 (34.3)	
• 2015-2019		108 (60.3)		69 (61.6)		39 (58.2)	
• 2020-2024		13 (7.3)		11 (9.8)		2 (3.0)	
Time interval between MRIs, years	179	2.1 (0.8)	112	2.1 (0.8)	67	2.0 (0.7)	0.97
Male sex, N (%)	179	99 (55.3)	112	80 (71.4)	67	19 (28.4)	<0.001
Age, years	179	38.2 (11.0)	112	36.4 (10.2)	67	41.3 (11.6)	0.004
Symptom duration, years	137	8.2 (9.2)	87	8.2 (8.4)	50	8.2 (10.7)	0.61
HLA-B27 positive, N (%)	173	114 (65.9)	110	87 (79.1)	63	27 (42.9)	<0.001
BASDAI	86	4.9 (2.4)	61	4.8 (2.5)	25	5.0 (2.2)	0.71
ASDAS	82	3.1 (1.1)	58	3.2 (1.1)	26	2.9 (0.9)	0.15
C-reactive protein, mg/l	91	11.4 (15.8)	65	13.1 (17.3)	26	7.2 (10.2)	0.004
Peripheral arthritis ever, N (%)	145	87 (60.0)	95	54 (56.8)	50	33 (66.0)	0.37
Enthesitis ever, N (%)	166	52 (31.3)	103	28 (27.2)	63	24 (38.1)	0.17
Dakytylitis ever, N (%)	175	17 (9.7)	109	11 (10.1)	66	6 (9.1)	1.00
EMM, N (%)	171	79 (46.2)	109	51 (46.8)	62	28 (45.2)	0.87
Current smoking, N (%)	177	58 (23.8)	111	36 (32.4)	66	22 (33.3)	0.98
Body mass index	141	25.4 (4.5)	86	24.9 (4.2)	55	26.1 (4.8)	0.16
MRI fulfilling ASAS definition, N (%)	179	87 (48.6)	112	87 (77.7)	67	0 (0.0)	<0.001
Radiographic axSpA, N (%)	76	21 (27.6)	49	17 (34.7)	27	4 (14.8)	0.20
SPARCC BME, median (IQR)	179	7.5 (1.5; 19.0)	112	14.5 (5.8; 27.0)	67	2.0 (0.3; 6.0)	<0.001
SPARCC Erosions, median (IQR)	179	3.0 (0.5; 7.3)	112	5.5 (2.5; 9.5)	67	1.0 (0.0; 1.5)	<0.001
SPARCC Fat, median (IQR)	179	1.5 (0.0; 5.5)	112	2.5 (0.0; 9.5)	67	0.0 (0.0; 2.5)	<0.001
SPARCC Backfill, median (IQR)	179	0.0 (0.0; 0.8)	112	0.0 (0.0; 2.0)	67	0.0 (0.0; 0.0)	<0.001
SPARCC Ankylosis, median (IQR)	179	0.0 (0.0; 0.0)	112	0.0 (0.0; 0.0)	67	0.0 (0.0; 0.0)	<0.002

Values are the mean (SD), except where indicated otherwise. ASDAS = Ankylosing Spondylitis Disease Activity Score; axSpA = axial spondyloarthritis; BASDAI = Bath Ankylosing Spondylitis Disease Activity Index; BME = Bone Marrow Oedema; EMM = Extra-musculoskeletal manifestations; HLA-B27 = human leucocyte antigen B27; MRI = Magnetic Resonance Imaging; OCI = osteitis condensans ilii. *No patients from Iceland fulfilled the inclusion criteria of this study.

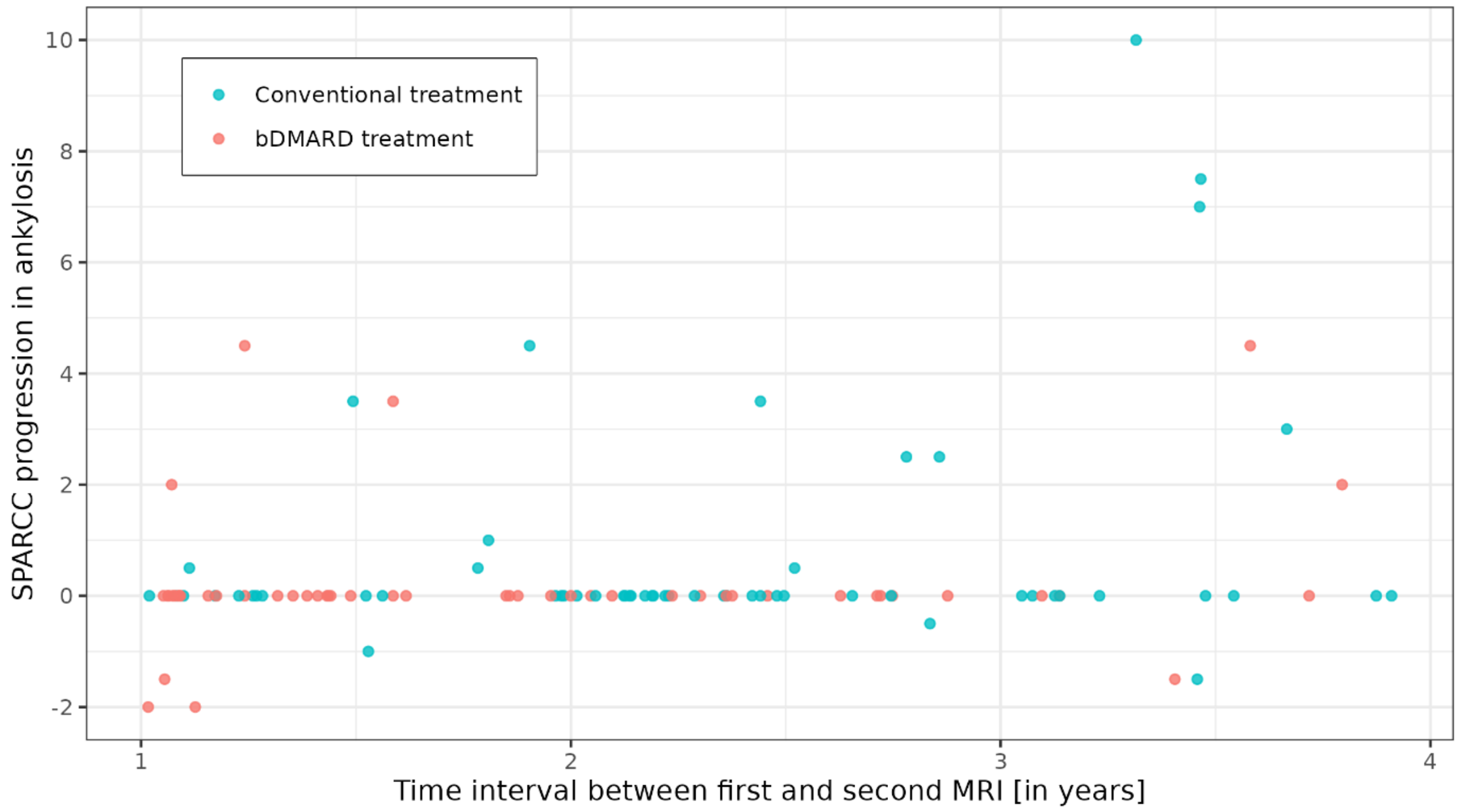

Supplemental Figure S1. Scatter plot illustrating SPARCC ankylosis progression scores in relation to the time interval between the first and second MRI scans in individual patients treated either with conventional therapy (red dots) or bDMARDs (cyan dots). Ankylosis progression is defined as an increase of at least 2 units in the SPARCC ankylosis sum score, exceeding the smallest detectable change of 1.4.

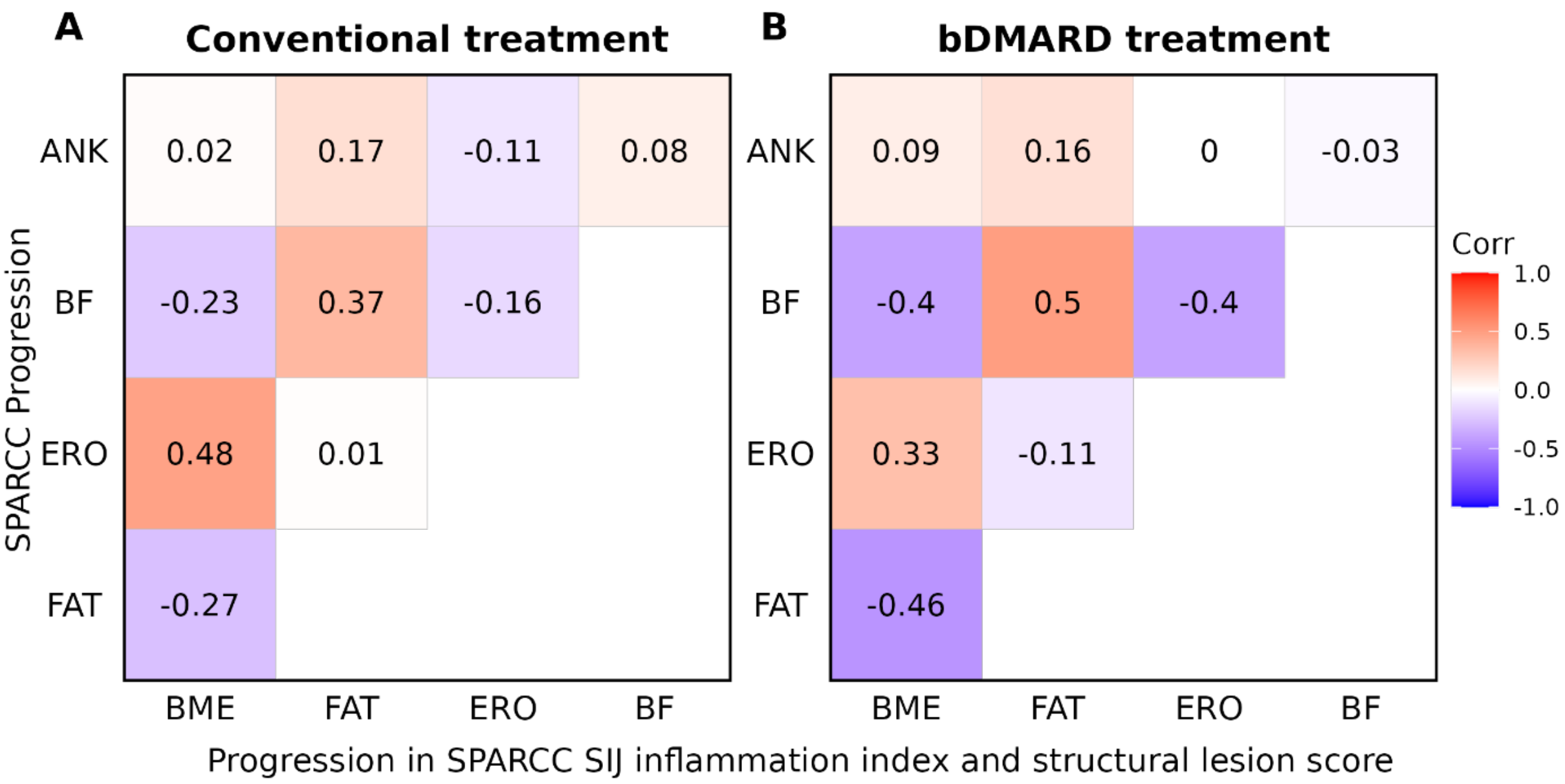

Supplemental Figure S2. Correlation matrix demonstrating associations between progression in SPARCC sacroiliac joint inflammation index and structural lesion score from baseline to follow-up on the x-axis and the progression of individual lesions on the y-axis. **A.** Correlation in patients on conventional treatment **B.** Correlations in patients treated with a first bDMARDs during the MRI interval. Spearman's rank correlation is used with the following interpretation of coefficients: 0 to 0.19 very weak association; 0.2 to 0.39 weak association; 0.4 to 0.59 moderate association; 0.6 to 0.79 strong association; 0.8 to 1 very strong association. ANK = ankylosis; axSpA = axial spondyloarthritis; bDMARD = biologic disease-modifying antirheumatic drug; BF = backfill; BME = bone marrow oedema; ERO = erosions; FAT = fat lesions; SCL = sclerosis. Conventional therapy refers to treatment with nonsteroidal anti-inflammatory drugs, analgetics and/or physical therapy.
